# Deep Learning–Based Survival Analysis Identified Associations Between Molecular Subtype and Optimal Adjuvant Treatment of Patients With Gastric Cancer

**DOI:** 10.1200/CCI.17.00065

**Published:** 2018-03-14

**Authors:** Jeeyun Lee, Ji Yeong An, Min Gew Choi, Se Hoon Park, Seung Tae Kim, Jun Ho Lee, Tae Sung Sohn, Jae Moon Bae, Sung Kim, Hyuk Lee, Byung-Hoon Min, Jae J. Kim, Woo Kyoung Jeong, Dong-Il Choi, Kyoung-Mee Kim, Won Ki Kang, Mijung Kim, Sung Wook Seo

**Affiliations:** **Jeeyun Lee**, **Ji Yeong An**, **Min Gew Choi**, **Se Hoon Park**, **Seung Tae Kim**, **Jun Ho Lee**, **Tae Sung Sohn**, **Jae Moon Bae**, **Sung Kim**, **Hyuk Lee**, **Byung-Hoon Min**, **Jae J. Kim**, **Woo Kyoung Jeong**, **Dong-Il Choi**, **Kyoung-Mee Kim**, **Won Ki Kang**, and **Sung Wook Seo**, Samsung Medical Center, Sungkyunkwan University School of Medicine, Seoul, Korea; and **Mijung Kim**, Ghent University, Ghent, Belgium.

## Abstract

**Purpose:**

Gastric cancer (GC) is the third-leading cause of cancer-related deaths. Several pivotal clinical trials of adjuvant treatments were performed during the previous decade; however, the optimal regimen for adjuvant treatment of GC remains controversial.

**Patients and Methods:**

We developed a novel deep learning–based survival model (survival recurrent network [SRN]) in patients with GC by including all available clinical and pathologic data and treatment regimens. This model uses time-sequential data only in the training step, and upon being trained, it receives the initial data from the first visit and then sequentially predicts the outcome at each time point until it reaches 5 years. In total, 1,190 patients from three cohorts (the Asian Cancer Research Group cohort, n = 300; the fluorouracil, leucovorin, and radiotherapy cohort, n = 432; and the Adjuvant Chemoradiation Therapy in Stomach Cancer cohort, n = 458) were included in the analysis. In addition, we added Asian Cancer Research Group molecular classifications into the prediction model. SRN simulated the sequential learning process of clinicians in the outpatient clinic using a recurrent neural network and time-sequential outcome data.

**Results:**

The mean area under the receiver operating characteristics curve was 0.92 ± 0.049 at the fifth year. The SRN demonstrated that GC with a mesenchymal subtype should elicit a more risk-adapted postoperative treatment strategy as a result of its high recurrence rate. In addition, the SRN found that GCs with microsatellite instability and GCs of the papillary type exhibited significantly more favorable survival outcomes after capecitabine plus cisplatin chemotherapy alone.

**Conclusion:**

Our SRN predicted survival at a high rate, reaching 92% at postoperative year 5. Our findings suggest that SRN-based clinical trials or risk-adapted adjuvant trials could be considered for patients with GC to investigate more individualized adjuvant treatments after curative gastrectomy.

## INTRODUCTION

Gastric cancer (GC) is one of the most frequently occurring malignancies worldwide and the third-leading cause of cancer-related deaths worldwide.^1^ Most patients with GC present with metastatic disease at recurrence, and the overall prognosis remains poor, with an expected survival of < 1 year upon recurrence. Several pivotal clinical trials were performed in the previous decade and aimed at reducing the recurrence rate after curative surgery in patients with GC. First, the Intergroup 0116 (INT-0116) trial published in 2001 demonstrated significant improvement in survival when patients with completely resected GC received postoperative chemoradiotherapy (CRT) with fluorouracil (FU) and leucovorin (LV).^2^ The Adjuvant Chemoradiation Therapy in Stomach Cancer (ARTIST) trial was a phase III trial that compared postoperative treatment with capecitabine plus cisplatin (XP) versus XP plus radiotherapy (RT) in patients with extended D2 lymph node dissection.^3,4^ The Capecitabine and Oxaliplatin Adjuvant Study in Stomach Cancer trial compared capecitabine plus oxaliplatin treatment with observation in completely resected GCs and demonstrated an additional survival benefit with adjuvant capecitabine plus oxaliplatin chemotherapy.^5^ The Adjuvant Chemotherapy Trial of Titanium Silicate for GC trial compared titanium silicate (TS-1) with observation in patients with D2-resected GC and also showed prolonged survival in the TS-1 chemotherapy group.^6^ Hence, there are at least three to four postoperative chemotherapy regimens available for patients with completely resected GC.

To additionally complicate clinical decision making, two recent molecular landscape studies demonstrated the existence of different molecular GC subtypes.^7,8^ The Asian Cancer Research Group (ACRG) categories were determined on the basis of gene expression profiling of tumors with microsatellite instability (MSI), tumors with an epithelial-to-mesenchymal transition phenotype, tumors with a *p53* signature (*CDKN1A* and *MDM2* expressing), or tumors without the *p53* signature. Notably, the ACRG study identified four distinct molecular subtypes highly associated with GC recurrence rate and, thus, survival after surgery.^8^ To the best of our knowledge, no model currently exists that is capable of integrating ACRG molecular subtypes and clinicopathologic information, such as stage, Lauren classification, type of surgery, demographic data, and molecular subtypes, to predict survival after surgery.

In this study, we developed a deep learning–based prediction algorithm for survival prediction in patients with GC on the basis of data from 1,190 patients with GC. The aims of this study were to develop a deep learning–based prediction model to predict survival after surgery on the basis of sequential prediction of the outcome at each time point until 5 years after operation and to predict the most optimal postoperative regimen after surgery using a recurrent neural network (RNN) on the basis of available clinical variables.

## PATIENTS AND METHODS

### Patients

We included the following three cohorts from our previous study: the ACRG cohort (n = 296),^8^ the postoperative FU/LV/RT cohort (n = 432; Gene Expression Omnibus database identifier: GSE26253),^9^ and the ARTIST cohort (n = 458).^3^ In total, 1,186 patients were included. We procured all tissue specimens that were chemotherapy naïve during the primary resection of GC. No patients received neoadjuvant chemotherapy or preoperative CRT. The following data were available for the three cohorts: pathology, type of surgery, lymphatic invasion, perineural invasion, histologic Lauren type, depth of invasion, number of dissected lymph nodes, number of positive lymph nodes (pathologically), age at diagnosis, sex, Epstein-Barr virus positivity, human epidermal growth factor receptor 2 positivity, adjuvant treatment modality, first sites of recurrence at the time of diagnosis for recurrence, date of recurrence, vital status at last follow-up, and molecular subtypes. Detailed information about these cohorts has been published previously.^8,9^ Briefly, 141 patients in the ACRG cohort received adjuvant chemotherapy or CRT. The ARTIST trial was a prospective adjuvant phase III trial that compared patients who received six cycles of XP (n = 228) with patients who received two cycles of XP followed by RT with capecitabine and then two more cycles of XP (n = 230). In the FU/LV/RT cohort, all patients (n = 432) received the INT-0116 regimen (FU/LV followed by CRT with the same agents and then FU/LV again separately) as an adjuvant postoperative treatment. All of the patients in the FU/LV/RT and ARTIST cohorts received adjuvant treatment after D2 resection. The overall survival rates after 5 and 10 years were 63.9% and 56.9%, respectively. Tumor recurrence was observed in approximately 40% of the patients for each cohort. The protocol was approved by the Samsung Medical Center Institutional Review Board (IRB; ACRG: IRB No. 2010-12-088), ARTIST (ClinicalTrials.gov identifier: NCT0176146), and the DASL (cDNA-mediated Annealing, S Selection, extension and Ligation) cohort (IRB No. SMC 2010-10-025).

### Molecular Subtypes

We used a previously published data set (accessed via https://www.ncbi.nlm.nih.gov/geo/query/acc.cgi?acc=GSE62254). RNA was extracted from 300 tumors according to manufacturer protocol (Affymetrix, Santa Clara, CA),^8^ and we used the Affymetrix human genome U133 Plus 2.0 array (Affymetrix) for gene expression profiling. The ACRG subtypes were the same as those published previously.

### Data Preprocessing

Primary data were specified according to their variable types. Categorical variables, such as sex, tumor type and location, and molecular expression, were converted using one hot encoding, which transforms categorical features to a binary (0 or 1) format group. Ordinal variables and quantitative variables were preprocessed with normalization. All variables were then transformed by a standard scaler to standardize the intervals of values between the variables. To save the numbers down to the third decimal place, all values were multiplied by 10^3^ and then transformed into integers (int32). As such, the value of each variable was transformed into a standard score, which was compatible with embedding. Missing data were imputed using the k-nearest neighbor algorithm after separating the training and test sets. The event cases during the time interval were ranked by months, and the rank scores were inserted in the censored cases.

### Data Separation and Cross-Validation

Patients were randomly sorted into a training set (80%) and a test set (20%). The test set was separated for the final test. Using the training set, bootstrap training (80% of the training set) and validation patients (20% of the training set) were generated by randomly selecting patients and repeating the selection 100 times to find an optimal condition of the neural networks. The validation error reached to the minimum at epoch 7. The optimized model was tested over the separated test patients.

### Performance Evaluation and Statistics

Receiver operating characteristics (ROC) curves, areas under the receiver operating characteristic curve (AUCs), and concordance index (c-index) were compared using a nonparametric Mann-Whitney *U* test using the MedCalc program (MedCalc Software, Seoul, Korea). All neural networks were constructed using the Keras with Theano backend in Python (https://keras.io/). The scikit-learn library (http://scikit-learn.org/) was used for data management and preprocessing. The Mann-Whitney *U* test was performed to compare AUCs between models, whereas the Pearson χ^2^ test was performed to identify factors related to a specific subgroup. The current study was developed and written according to Transparent Reporting of a Multivariable Prediction Model for Individual Prognosis or Diagnosis model development guidelines.

### Basic Concept of Survival Recurrent Network Model

A survival recurrent network (SRN) model was constructed on the basis of logistic regression function σ at discrete time point (*t*) and long short-term memory (LSTM) neural network,^10,11^ which can be represented as follows:

$$Survival\;probability\;at\;time\;\left(t\right):\;\;ft(X)=\sigma \left(\;Wt*Xt\right)=\;\frac{1}{1+e^{-\omega_{t}X_{t}}}$$

$$HRt(X)=e^{W_{t}*X_{t}}$$

$$HRt(X)=e^{\theta_{1}X+\theta_{2}t}$$

where ft(X)is the survival probability, HRt(X)is the hazard ratio function.

Once the parameter vector, *W_t_* = θ_1_*X* + θ_2_*t*, is optimized with the patient group (features *X_t_* and target *Y_t_*) at the first time point (*t*), LSTM cells memorize *W_t_* and the model is retrained with the target value (*Y_t_*_+1_) of the next time point yielding *W_t_*_+1_. For example, if a patient (*X*) died of disease 2 years after the first visit, the model should learn the target value (*Y*_1_ = 1) at the first year and learn the target value (*Y*_2_ = 0) at the second year. Because LSTM memorizes and optimizes *W* for each target value_,_ our RNN-based model is able to infer a target value at a certain time point.

Patient factors (*X*) were hardly updated at every time point because we could not collect all those data without any loss. Moreover, the purpose of the survival model is to predict long-term survival with the information from the first visit. Thus, we generated *X_t_* with the following assumption: patients’ features should be constant during the observation time. Instead, there should be latent features that are dependent to the sequential time and indicate the patients’ status at a discrete time point. We defined those latent features as time-dependent life value:

$$Time‐dependent\;life\;value=\theta_{2}\text{t}+\theta_{3}\partial \text{S}$$

$$Time‐dependent\;hazard\;function\;\;e^{WtXt}\;=e^{\theta_{1}X+\theta_{2}t+\theta_{3}\partial S}$$

The life value dimensions were embedded in the constant patient features (*X*) to generate time-dependent features (*X_t_*). The life value features consisted of time (*t*) and prior life expectancy (*S_t_*), which is updated using gradient descent equation (∂S).

The model is retrained at the following sequential time with *X_t_*_+1_ where *S_t_*_+1_ is embedded.

St+1=St + ∂S

$$St+1=St\;+\;\alpha \left(Yt-\;\hat{Y}t\right)\frac{ft^{'}\left(x\right)}{\left|ft\left(x\right)\right|}*\hat{Y}t$$

$$\frac{dft(x)}{dx}=-\text{ W*}\frac{e^{-wx}}{{\left(1\;+\;e^{-wx}\right)}^{2}}\approx \;\alpha *ft(x)(1-ft\left(x\right))\;$$

where $\text{α is a step size and }\hat{Y}t\;=ft(x)$. Thus, the function can be rewritten as follows:

$$St+1=St\;+\;\alpha (Yt-\;\hat{Y}t)(1-\hat{Y}t)\hat{Y}t$$

## RESULTS

### Predictive Accuracy of the SRN Model

**Fig A1.**
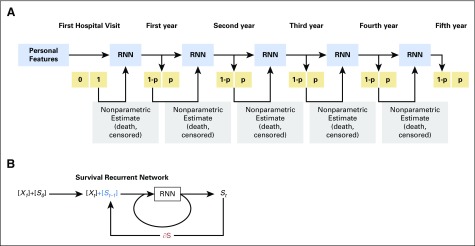
Survival recurrent network (SRN) schema. (A) SRN simulated the sequential learning process of clinicians in the outpatient clinic using a recursive neural network (RNN) and time-sequential outcome data. The basic learning unit was composed of the RNN system and analyzes patient information at the first visit. At each time point, the unit takes this prediction and trains itself according to the actual survival data. If patients were censored during the time interval, the ranking scores estimated among the censored patients are used for training instead of survival data. The probability of survival is input and learned for the prediction of the survival probability for the following year. This sequential loop ends at the 5-year visit to yield the final survival probability. (B) Schematic SRN equation. The life value features consisted of time (*t*) and prior life expectancy (*S_t_*), which is updated using the gradient descent equation (∂S). The model is retrained at the following sequential time with *X_t_*_+1_ where *S_t_* is embedded.

**Fig A2.**
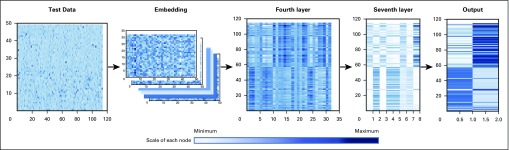
The architecture of the basic survival recurrent network learning unit. The network architecture of the basic unit comprised eight hidden layers with a single recursive neural network layer (long short-term memory). The input layer comprised 49 nodes that represented 47 input features and two survival features. The output layer comprised two nodes implementing the Softmax function, which represents live or dead probability. The individual features were embedded in a 49 × 32 vector. The other four layers comprised fully connected nodes implementing the rectified linear-unit function. The number of nodes was gradually reduced across the hidden layers.

**Fig A3.**
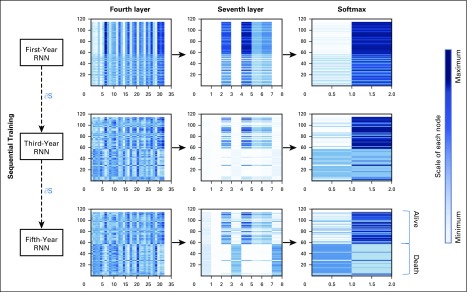
The architecture of the survival recurrent network (SRN) time-sequential learning unit. The SRN was trained every year sequentially with the 47 clinical features and the previous survival probability. Through this time-sequential training, the probability difference among patients became more distinct, and the accuracy improved. RNN, recursive neural network.

We simulated the sequential learning process of clinicians in the outpatient clinic using RNN and time-sequential outcome data (Appendix Fig A1A, online only). The SRN was composed of the following three learning system parts: the first included information on patient status (covariates, *X*), the second included the time-dependent life value ($\theta_{2}$*t*+$\theta_{3}\partial$S), and the third included nonparametric rank scores (R) of event that occurred during the interval (0 < R < 1). The R was inserted into the target value instead of binary values. The *X_t_* were input into the SRN, and the SRN was sequentially retrained with the updated *X_n_* (Appendix Fig A1B). The network architecture of the SRN comprised five hidden layers with two RNN layers (LSTM). The input layer comprised 49 nodes that represented 47 input features and two life value features (phase [time] feature and prior survival probability feature). The output layer comprised two nodes implementing the Softmax function, which represents live or dead probability. Forty-seven features were preprocessed before accessing the input layer, with each feature preprocessed using a standard scaler and each value encoded as an integer within 10,000 scores. The clinical variables of an individual were embedded in a 47 × 32 vector for dimensionality reduction. The other two layers comprised fully connected nodes implementing the rectified linear-unit function. Gaussian dropout was performed to prevent overfitting problems. The number of nodes was gradually reduced across the hidden layers (Appendix Fig A2, online only). The SRN was trained every year sequentially with the 47 clinical features and the previous survival probability. Through this time-sequential training, the probability differences among patients became more distinct, and the accuracy improved (Appendix Fig A3, online only).

We randomly sorted 1,186 patients into a training group (80%; n = 950) and a test group (20%; n = 236). In the training group, bootstrap training (80%; n = 760) and validation populations (20%; n = 190) were generated by randomly selecting patients and repeating the selection 100 times. At each time point, the predicted survival probability of the model was compared with the actual survival data.

**Fig 1.**
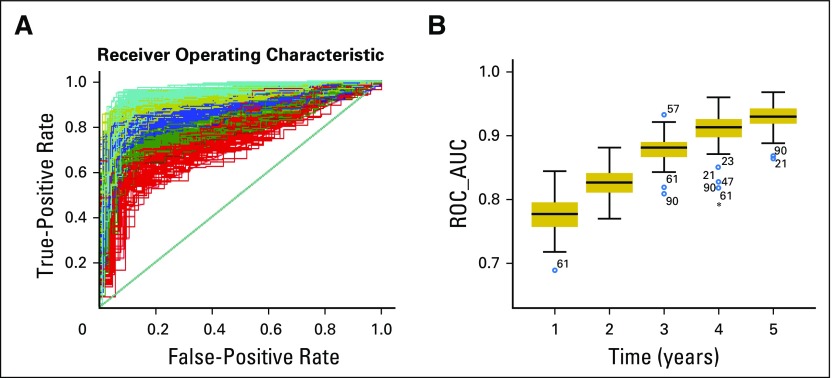
The mean receiver operating characteristic (ROC) curve for 100 validation data sets. (A) The 100 trained survival recurrent networks (SRNs) were tested iteratively using the corresponding test patients, and ROC curves were generated to evaluate the average predictive power at each time point. (B) The mean area under the ROC curve (AUC) of the test data sets. The AUC of each time point was compared using the Mann-Whitney *U* test. The AUC of the fifth year was significantly higher than that of the second year (*P* = .00) or the first year (*P* = .00). The AUC of the fourth year was significantly higher than that of the second year (*P* = .00) or the first year (*P* = .00).

The mean AUC of the 100 training group patients was 0.79 ± 0.052 at the first year, 0.839 ± 0.045 at the second year, 0.89 ± 0.049 at the third year, 0.915 ± 0.05 at the fourth year, and 0.92 ± 0.049 at the fifth year (Fig 1A). The AUC of each time point was compared using the Mann-Whitney *U* test, showing that the AUC improved significantly in sequential years. The AUCs at the fifth and fourth years were significantly higher than those at the first or second years according to the SRN model (Fig 1B).

### Performance of the Final Model for Predicting Survival of Sample Test Group

**Fig 2.**
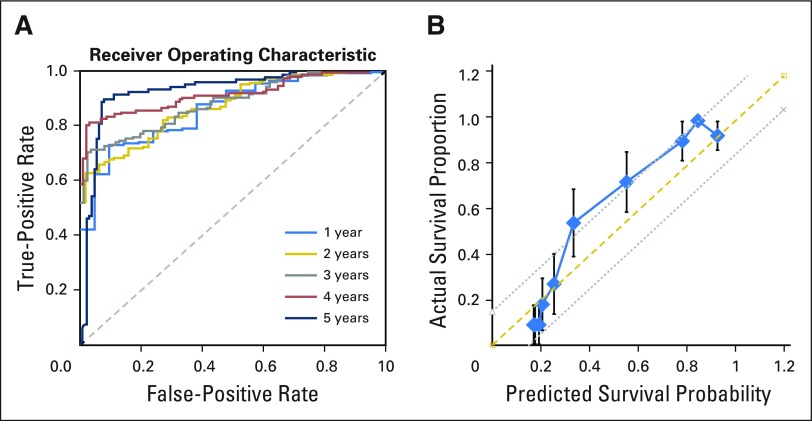
The receiver operating characteristic (ROC) curves and the calibration plot of the survival recurrent network (SRN) for predicting survival of a sample test group. (A) The ROC and area under the ROC curve of the final model were evaluated using the separated test group at each 5-year point. (B) Calibration curve of the SRN in the test data set. The *x*-axis represents the probability of 5-year survival, and the *y*-axis represents the actual survival proportion. The thick solid line represents the calibration plot of the SRN. The actual survival proportion and 95% CIs were calculated using the Kaplan-Meier method. The prediction scores were within a 15% margin of the perfect prediction line (dashed line).

Using the separated test group, the performance of our final model was evaluated. The AUC of each time point was 0.858 at the first year, 0.869 at the second year, 0.879 at the third year, 0.912 at the fourth year, and 0.923 at the fifth year (Fig 2A).

The c-index of the final model was evaluated with the test group. The c-index was 0.951.

The calibration curve from the SRN was evaluated using the sample test group (Fig 2B). In this curve, the actual survival proportion was compared with the SRN-predicted survival probability, with the actual survival rate calculated using the Kaplan-Meier method. Our results showed that the actual survival rate was closely correlated with the predicted survival probability within 15% margin of error.

**Fig 3.**
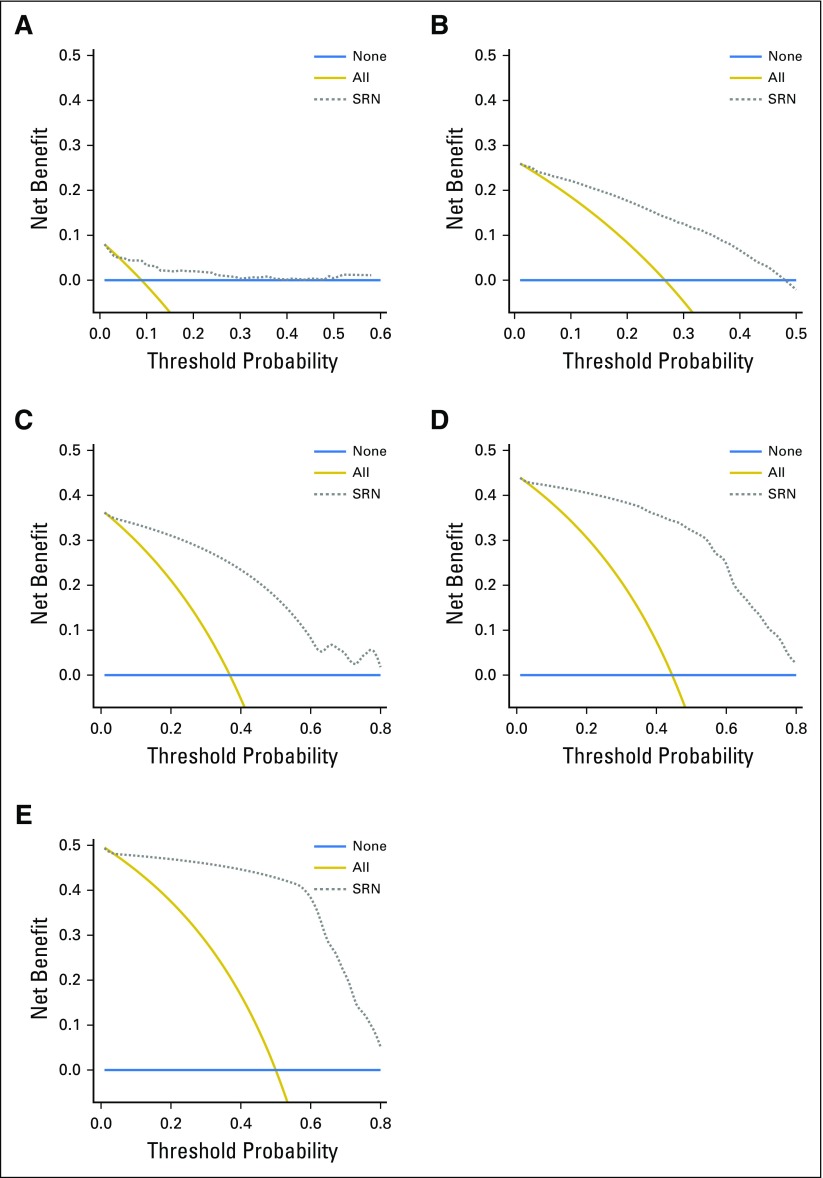
The decision curve analysis (dashed line) of the survival recurrent network (SRN) model for predicting the survival of sample test group. The prediction model has a positive net benefit if the line is above the lines assuming none of the patients or all of the patients survive at each year. (A) First year. (B) Second year. (C) Third year. (D) Fourth year. (E) Fifth year. The decision curves of the model show positive net benefit rather than assuming that all patients or none of the patients will survive at each year.

The decision curves showed that the SRN model will be useful clinically for predicting the survival of patients with GC. The SRN model has a positive net benefit at all five different time points, rather than assuming all patients or none of the patients will survive at each year (Fig 3).

### Cumulative Survival Probability of Individual Patients in the Test Group

**Fig A4.**
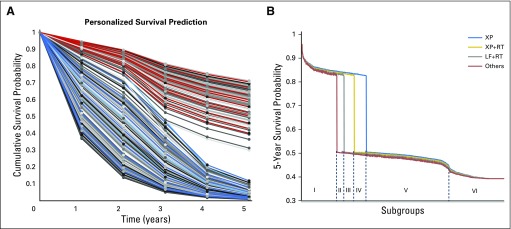
(A) Cumulative survival probability of a sample test group. The survival recurrent network (SRN) survival prediction curve was unique for each individual according to their unique clinical inputs. Two clusters were distinctly divided until the fifth year (blue lines indicate poor prognostic group; red lines indicate good prognostic group). (B) Five-year survival probability graph after simulation of four different adjuvant treatments by the trained SRN. The trained SRN was used to predict the survival probability associated with a virtual data set, in which four different adjuvant options were applied to all patients. According to the probability, the following distinct subgroups were identified: subgroup I, good prognostic subgroup regardless of adjuvant therapy; subgroup II, good responder to three adjuvant options (cisplatin [XP], XP plus radiotherapy [RT], and leucovorin-fluorouracil [LF] plus RT); other options, including no treatment, abruptly decreased the survival probability < 50%; subgroup III, good responder to XP and XP plus RT, although LF plus RT should not be recommended because the response to LF plus RT was poor in this subgroup; subgroup IV, good responder only to XP, with XP without RT highly recommended in this subgroup; and subgroups V and VI, poor prognostic subgroups, where conventional adjuvant options will not be effective. In subgroups V and VI, trial regimens should be initiated from the beginning.

The cumulative survival probability of each patient was visualized as a survival graph. After a sequential learning process, two clusters of patients were separated in terms of survival curves around the postoperative fifth year (Appendix Fig A4A, online only). To identify significant factors differentiating the two prognostic clusters, we performed the Pearson χ^2^ test. Positive perineural invasion, the presence of lymphovascular invasion, high tumor or node stage, pathologic stage, a primary tumor located in the antrum or cardia, and recurrence were more frequently observed to be statistically significant in the poor prognostic group in terms of survival (Table 1).

**Table 1.**
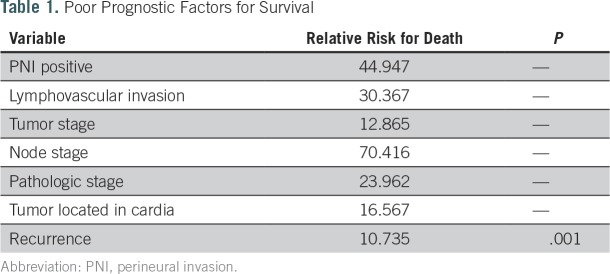
Poor Prognostic Factors for Survival

### SRN Guidance of the Optimal Adjuvant Treatment Regimen

To determine the optimal postoperative regimen according to the available data for each patient, we generated a new simulation data set, in which four different treatment options were applied to all patients. Five subgroups were identified according to the survival probability at the fifth year (Appendix Fig A4B). The treatment options included in the analysis were as follows: XP chemotherapy; XP followed by capecitabine plus RT followed by XP; the INT-0116 regimen; and others, such as oral TS-1 chemotherapy. Because oral chemotherapy or other regimens composed < 5% of the options, we excluded this population from additional analysis. For all adjuvant treatments, significant predictors for poor responders in terms of survival after adjuvant treatment were mesenchymal subtype, the presence of perineural invasion, advanced stage, location of the primary tumor in the cardia, and a greater number of positive lymph nodes (Table 2). Notably, we found that patients with GCs with MSI (odds ratio, 4.2; *P* = .042) and with GCs of the papillary type (OR, 5.9; *P* = .015) had significantly better survival outcomes after XP chemotherapy alone (Table 3). Subgroup categorization according to SRN resulted in the following five major groups (Fig A4B): subgroup I, good prognosis regardless of the adjuvant regimen; subgroup II, better prognosis after adjuvant chemotherapy, except for the others group; subgroup III, better prognosis after adjuvant XP plus RT and XP alone; subgroup IV, better prognosis after XP alone; and subgroup V, poor prognosis regardless of the type of postoperative regimen.

**Table 2.**
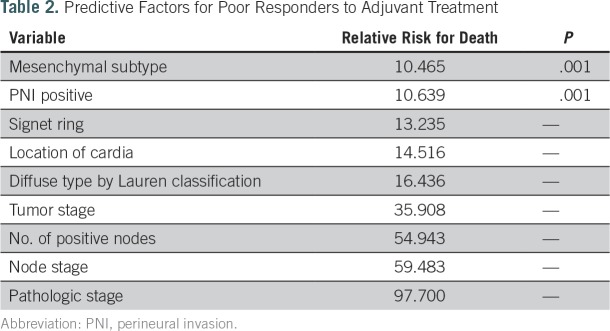
Predictive Factors for Poor Responders to Adjuvant Treatment

**Table 3.**
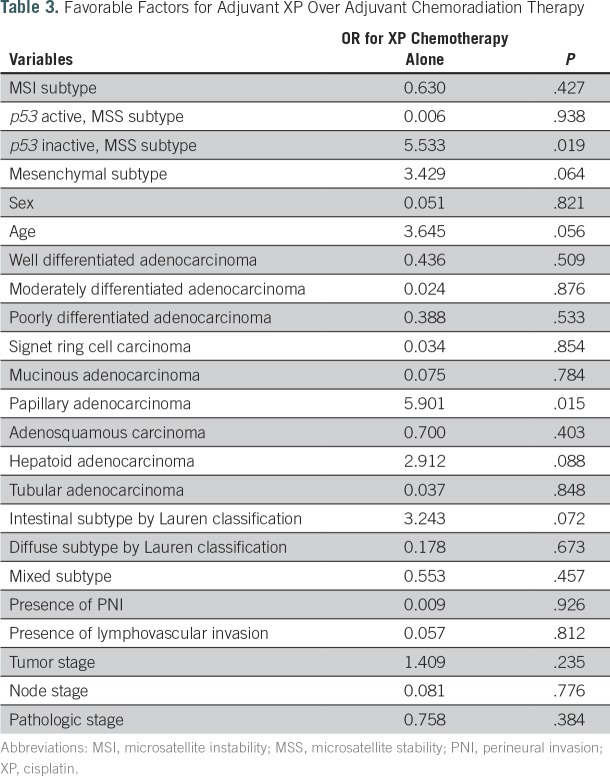
Favorable Factors for Adjuvant XP Over Adjuvant Chemoradiation Therapy

## DISCUSSION

Studies have recently been performed to formalize risk prediction in cancer care. Kaplan-Meier curves represent a nonparametric method for estimating survival, with one of the strengths of this method being its ability to consider censored data, particularly right-censoring data using the log-rank test. Parametric survival models and the Cox proportional hazards model might be useful to estimate covariate-adjusted survival. The Cox proportional hazards model is one of the most popular methods for predicting cancer prognoses. A recent Cox model for survival prediction of patients with GC showed an average c-statistic of 0.822.^12^ The Cox model uses the hazard rate of the covariates as model coefficients and, therefore, can encompass factors that change over time. However, the model assumes that the hazard function is constant through patient life span; therefore, it cannot accurately predict the risk of death at a certain time point because it does not represent the changing weight of covariates during the time intervals. A parametric model, such as a linear regression model, measures a direct relationship between the covariates and the survival time. If the data from the survival distribution at each time point are not large enough or if there are large numbers of missing values, the survival distribution at each time point becomes unreliable. This model often produces misleading outcomes in the event of a violation of the proportionality of the hazard assumption. Significant risk factors that are not time dependent are often ignored in these models, thereby potentially resulting in false inferences. Another cause of misleading results is that the residual, the difference between the real and expected values, is not properly reflected in the model.^13^ Another recent model, the least absolute shrinkage and selection operator, became a popular regression method as a result of its enhanced predictive accuracy acquired by shrinking important covariates from thousands of variables, especially when using genetic data.^14^

Our model simulated the physician learning process. Physicians learn about the prognoses of their patients through serial observations in outpatient clinics. At the first visit, a clinician predicts the patient condition at the next visit on the basis of their current medical status and confirms that prediction during the subsequent visit. We suggest that LSTM, an RNN proposed in 1997 by Sepp Hochreiter and Jürgen Schmidhuber,^10,11^ represents the optimal choice for a serial learning system. LSTM was designed to avoid the long-term dependency problem of RNNs and comprises input, output, and forget gates, which determine whether a value should be remembered or forgotten. Because LSTM can remember a value for a longer time period (ie, it is iterative), it is useful for training using clinical data with various time series. However, LSTM exhibits a limited ability to estimate survival involving censored events. In our SRN model, the survival distribution at the discrete time points was determined by the logistic hazard function, in which death events were also scored relative to censored data. In every sequential time, the model was updated by the time-specific *X_t_*, which contains the first visit information, phase dimension, and survival probability dimension.

On the basis of the use of this algorithm, strong predictors for poor responders in terms of survival after adjuvant treatment were mesenchymal subtype, the presence of perineural invasion, advanced stage, location of the primary tumor in the cardia, and a greater number of positive lymph nodes. These findings supported previous studies that observed that mesenchymal subtype produces continuous recurrence and, thus, earlier death, after a 5-year surveillance program after surgery.^8^ Our SRN model was based on data from > 1,000 patients with GC and demonstrated that GC with a mesenchymal subtype located in the cardia should elicit a more risk-adapted postoperative treatment strategy. Interestingly, we found that GCs with MSI and GCs of the papillary type have significantly more favorable survival outcomes after XP chemotherapy alone. These factors will be validated in the ARTIST-II trial currently recruiting patients to compare TS-1 alone versus TS-1 and oxaliplatin versus TS-1, oxaliplatin, and RT in patients with D2-resected GC.

In this study, the available input data used for survival prediction were mainly pathologic results, molecular subtypes, adjuvant treatments, and recurrence information. However, there are other factors affecting patient survival that could not be evaluated in our study. These included accompanying comorbidities or nutritional status; operative factors, such as the amount of intraoperative blood loss, transfusion, or postoperative complications; and postoperative recovery factors, such as nutritional status, weight loss, or anemia.^10,11,15-17^ Therefore, although pathologic and molecular data are strong prognostic factors, they are not modifiable, but are merely predictive of survival and act as reference factors for deciding adjuvant treatment modality. Notably, some patient and operative factors can be modified and improved by doctor-patient deliberations. If our model can include these modifiable factors for each patient, more individualized treatments and accurate survival prediction would be possible. Another possible limitation of this study was that our model did not combine imaging data, which would be an excellent and natural extension of the current deep learning SRN approach.

In conclusion, our SRN predicted survival at a high rate, reaching 92% at postoperative year 5. SRN-based clinical trials or risk-adapted adjuvant trials should be considered for patients with GC to investigate more individualized adjuvant treatments.
